# Efficacy and Safety of Donanemab for Alzheimer's Disease: A Systematic Review

**DOI:** 10.1002/alz70859_103173

**Published:** 2025-12-25

**Authors:** Mariam M. Fouad, Nadeen A Nagy, Noreen M. El‐Bayaa, Nada A. Hamdan, Youssef A. Ismail

**Affiliations:** ^1^ Faculty of Medicine Port Said Univeristy, Egypt, Port Said, Port Said Egypt

## Abstract

**Background:**

Alzheimer's disease is a progressive neurodegenerative disorder that affects memory, thinking, and behavior, leading to a decline in cognitive function over time. This study aims to investigate the efficacy and safety of Donanemab to improve cognitive and behavioral symptoms in people living with Alzheimer's disease.

**Methods:**

We systematically searched in Cochrane, PubMed, Web of Science and SCOPUS since date of inception until Dec 1^st^ 2024. Due to limitations in the available data, a meta‐analysis was not conducted for this study. Instead, a synthetic approach was utilized to summarize and interpret the findings.

**Results:**

We initially identified 477 studies through database search and included only five studies in the systematic review, included studies showed a total of 2205 participants (1249 females, 56.64 %), 1133 participants received the drug and 1072 received placebo. The mean age ranged from 66.8 to 78.3 years. After dosing, a marked positive change of brain amyloid level without significant reaccumulation for up to 72 weeks was observed among patients receiving three to five doses of 10 mg/kg Donanemab. No significant amyloid reduction was seen in the lower Donanemab multiple dose levels (0.3 to 3mg/kg). To assess the efficacy and adverse effects of donanemab, we observed a significant increase in musculoskeletal disorders, specifically arthralgia, in both the intervention and control groups. Cerebral microhemorrhage showed a notable rise, predominantly in the intervention group compared to the control. Similarly, superficial siderosis increased markedly in the intervention group. Additionally, fatigue, falls, and headaches were reported with a marked increase across both groups at varying doses.

**Conclusion:**

Donanemab demonstrated a rapid, robust, and sustained reduction in brain amyloid plaque with no significant reaccumulation observed at 72 weeks. It was generally well‐tolerated at doses up to 10 mg/kg; however, its use is associated with significant adverse effects, highlighting the need for careful risk‐benefit evaluation.

